# Primary follicular dendritic cell sarcoma of the kidney – a case report of a rare tumor with emphasis on diagnostic pitfalls

**DOI:** 10.1186/s13000-024-01444-x

**Published:** 2024-01-31

**Authors:** Tamás Pancsa, Borbála Dénes, Áron Somorácz, Dóra Kelemen, Ferenc Salamon, Fanni Sánta, Levente Kuthi

**Affiliations:** 1 Department of Pathology, Albert Szent-Györgyi Medical School, University of Szeged, Állomás Street 1, Szeged, 6725 Hungary; 2 Medserv Ltd, Budapest, Hungary; 3 Pathology Unit, Uzsoki Street Hospital, Budapest, Hungary

**Keywords:** Follicular dendritic cell sarcoma, FDCS, Renal tumor, Differential diagnosis

## Abstract

**Background:**

Follicular dendritic cell sarcoma (FDCS) is a rare low-grade tumor of the lymph nodes, but roughly one-third of the cases emerge from extranodal sites, posing diagnostic challenges.

**Case presentation:**

In this report, we present the case of a 59-year-old lady who complained of renal colic. During investigation, a kidney tumor was discovered. A radical nephrectomy was performed, and histological examination identified the tumor as a sarcomatoid renal cell carcinoma. The case was then referred to a genitourinary pathologist for further evaluation. The tumor cells exhibited positive staining for CD21, CD23, somatostatin receptor 2 A, and MDM2 expression. Additionally, *MDM2* gene amplification was confirmed by the FISH study. Ultimately, the tumor was diagnosed as a primary renal FDCS. The patient was placed under active oncological surveillance and did not receive any further therapy. Remarkably, after 91 months of follow-up, she remains tumor-free.

**Conclusion:**

This case represents a well-documented primary renal FDCS. Our aim in presenting this extremely rare tumor is to enhance awareness and highlight the importance of considering FDCS in the differential diagnosis.

**Supplementary Information:**

The online version contains supplementary material available at 10.1186/s13000-024-01444-x.

## Background

Follicular dendritic cell sarcoma (FDCS) is a rare malignant tumor arising from follicular dendritic cells, essential for lymphoid follicle microarchitecture, B cell migration, and antigen presentation [1]. While most FDCS cases originate in lymph nodes, approximately 30% occur extranodally, affecting sites such as the head, neck, and gastrointestinal tract [2]. The classic FDCS typically consists of ovoid or spindle-shaped cells with small nuclei, eosinophilic cytoplasm, and inconspicuous cell borders [3]. Although rare, an epithelioid morphology can also be observed. Tumor cells may form bundles, solid sheets, fascicles, or storiform patterns [4]. A notable feature is the abundant lymphocytic infiltration among tumor cells [3, 4]. Additionally, some FDCS cases exhibit an inflammatory pseudotumor-like feature, marked by EBV genome positivity, exclusive to the spleen and liver [5]. Immunohistochemically, FDCS expresses usual markers of normal follicular dendritic cells, including clusterin, podoplanin, CD21, CD23, CD35, and CXCL13 [6]. Importantly, tumor cells are negative for CD1a, CD20, CD31, CD34, cytokeratin, EMA, and melanocytic markers [1–4]. The genetic background of FDCS remains poorly understood, with complex chromosomal losses and activation of the NFkB pathway reported, but no recurrent abnormalities identified to date [7]. Diagnosing FDCS can be challenging due to its diverse morphology and rarity, especially when presenting in atypical locations. This paper presents an unusual FDCS case originating in the kidney parenchyma. Furthermore, we summarize the key features of our case and highlight potential diagnostic pitfalls and differential diagnostic considerations.

## Case presentation

**Fig. 1:**
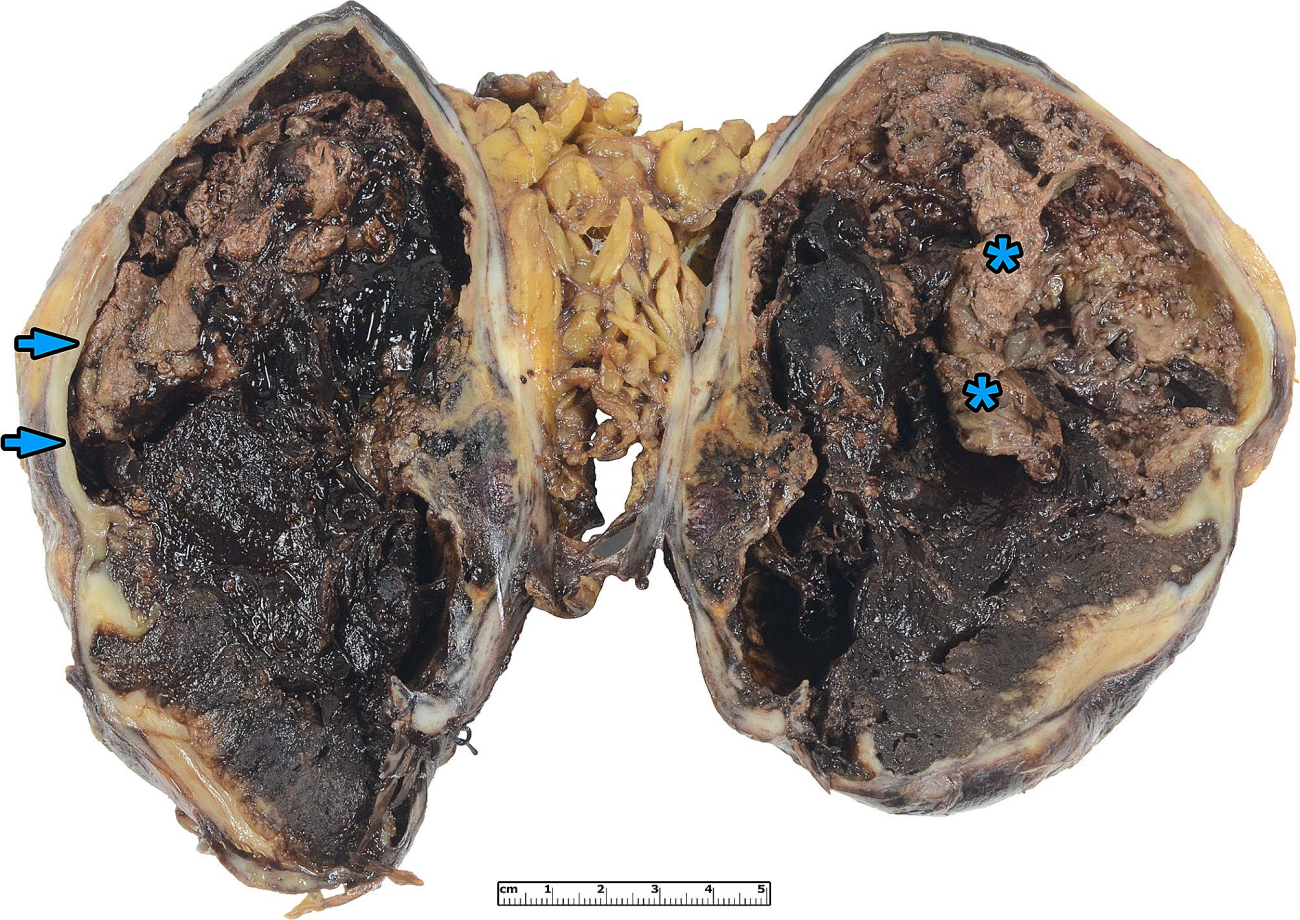
Macroscopic appearance of the renal follicular dendritic cell sarcoma. The tumor infiltrated the entire kidney, and an atrophic rim of the renal parenchyma was visible around the tumor (arrowheads). The tumor exhibited a hemorrhagic cut surface with focal solid areas in brown color (asterisk)

**Fig. 2:**
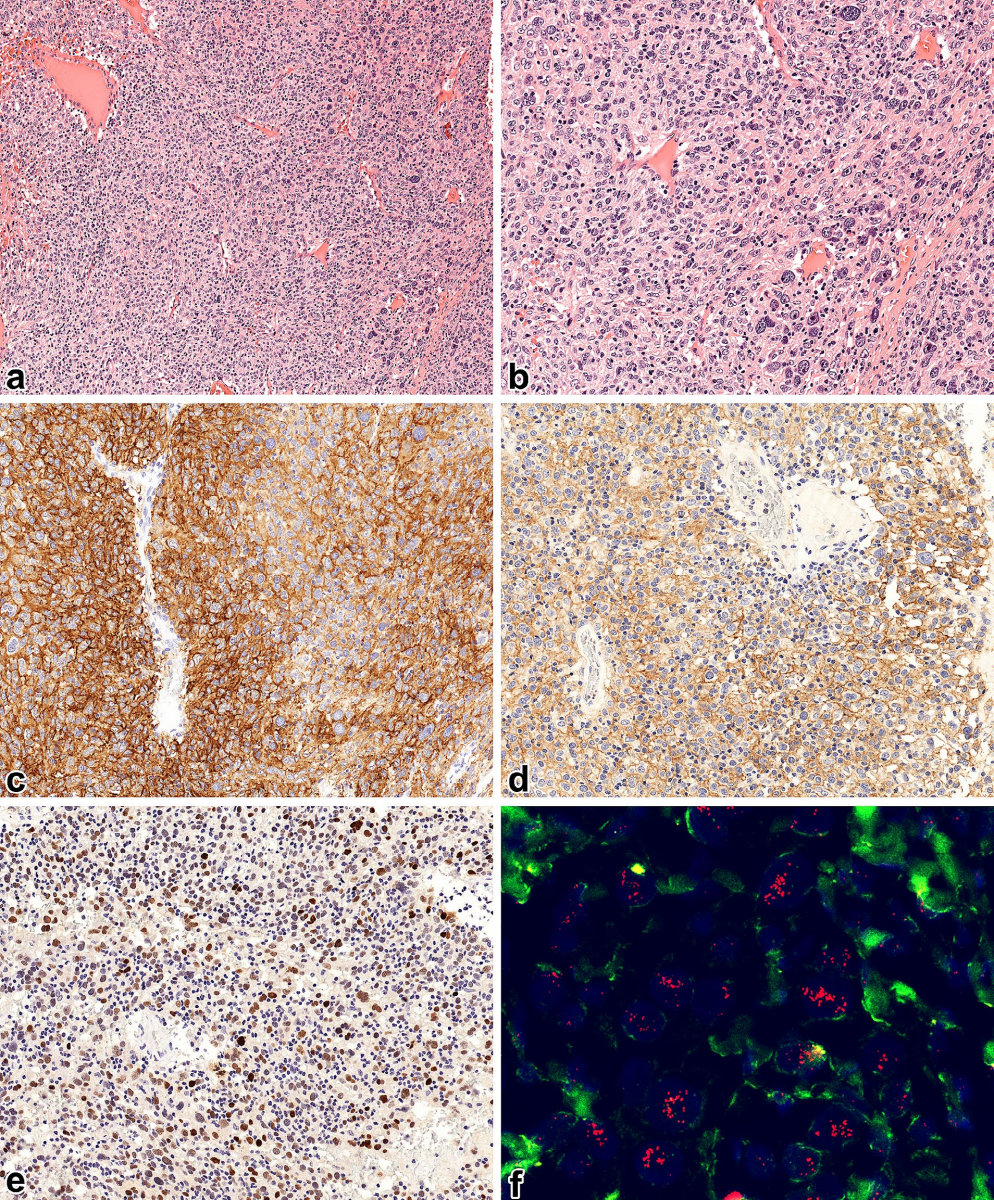
Microscopic features of the renal follicular dendritic cell sarcoma. **a** The neoplastic cells formed syncytial sheets with indistinct cell borders. One of the most characteristic findings was the striking lymphocytic infiltration throughout the tumor. The nuclei of the tumor cells showed grooves and lobulation, vesicular chromatin, and prominent nucleoli. The pleiomorphism was focally marked (magnification factor of 200x). **b** Despite the pleiomorphism, the mitotic rate was relatively low (magnification factor of 400x). **c** The neoplastic cells showed diffuse, strong positivity with CD21 (magnification factor of 200x). **d** The tumor cells displayed diffuse and moderately strong membranous staining with somatostatin receptor 2 A (magnification factor of 200x). **e** The neoplastic cells expressed MDM2 strongly and diffusely. This finding could lead to an erroneous diagnosis of dedifferentiated liposarcoma, especially in a case of a retroperitoneal tumor (magnification factor of 200x). **f** The *MDM2* FISH analysis demonstrated more than five *MDM2* (red) signals in the tumor cells, indicating gene amplification (magnification factor of 1200x)

A 59-year-old lady presented to our hospital with complaints of right renal colic. She had no significant medical history and denied any prior episodes of hematuria. Her laboratory parameters were within the normal range. On physical examination, tenderness in the right lumbar region was noted, but no palpable mass was detected. An abdominal ultrasound revealed no renal or ureter stones, but it identified a large 135 mm hyperechoic tissue mass in the kidney parenchyma. A contrast-enhanced computed tomography scan confirmed the presence of a right renal tumor, invading the entire kidney parenchyma but with no extrarenal infiltration. No lymphatic or hematogenous metastasis was found, leading to a decision for radical nephrectomy. During surgery, there was a complication with a 17 mm long rupture of the inferior vena cava, requiring four units of packed red blood cells and subsequent admission to the intensive care unit. The post-operative period was uneventful. Grossly, we investigated a 170 × 130 × 100 mm large, relatively well-defined tumor with hemorrhagic and solid areas (Fig. 1). Microscopically, the lesion was made up of epithelioid cells with eosinophilic cytoplasm forming bundles, fascicles, and storiform patterns. Adjacent to the tumor cells, small lymphocytes were observed (Fig. 2a). The tumor exhibited increased cytological atypia and a few (1 per 10 high-power field) mitotic figures (Fig. 2b), but no necrotic areas were seen. The initial diagnosis was a sarcomatoid renal cell carcinoma (RCC), and due to its unusual histological appearance, an immediate consultation with a genitourinary pathologist was sought. Further immunohistochemical studies showed the tumor cells were positive for CD21, CD23, somatostatin receptor 2A, and MDM2 (Fig. 2c-e), while negative for HHV8, PAX8, PAX2 AE1/AE3, CK7, MelanA, S100, CD68, SMA, TdT, desmin, and podoplanin (Suppl. Figure 1.). In addition, the dilution, clone and source of the antibodies applied are listed in Supplementary Table 1. An *MDM2* fluorescent in situ hybridization (FISH) revealed an *MDM2* gene amplification (Fig. 2f), while an Epstein-Barr Encoded RNA probe returned negative results. Finally, the diagnosis of primary renal follicular dendritic cell sarcoma with FNCLCC grade 2 and AJCC pT4 stage was established. The circumferential and hilar resection lines were free of tumors. The post-operative staging investigations showed no residual tumor or metastasis. The patient did not receive any adjuvant therapy and has been followed for 91 months since the initial presentation, during which she remains alive and disease-free.

## Discussion

FDCS is a rare mesenchymal tumor arising from the follicular dendritic cells [1–3]. The tumor is composed of spindle to ovoid cells having an immunophenotype similar to the normal follicular dendritic cells of the lymph nodes [8]. Nevertheless, around the tumor cells, an extensive inflammatory cell infiltration is present [1–4]. FDCS has a nodal form that was described in 1986, while its extranodal version was characterized in 1994 [9, 10]. In addition, extranodal FDCS most frequently develops in the head and neck region, the gastrointestinal tract, and the liver and spleen [1, 5, 8]. Currently, there are no known risk factors, but approximately 20% of the FDCS cases develops on the ground of hyaline-vascular Castleman disease [11]. Regarding the urinary tract, there are reports of FDCS arising in the urinary bladder and kidney parenchyma [12]. Misdiagnoses of extranodal FDCS cases remain common, with this case initially signed out as a sarcomatoid renal cell carcinoma, the primary entity in the differential diagnosis for this location. Although there is no kidney-specific immunohistochemical marker, PAX8 is a reliable test to confirm renal origin [13]. However, PAX8 is also expressed in other tumors, including thyroid, ovarian surface epithelial, neuroendocrine tumors, and lymphomas [14]. While PAX8 is generally negative in mesenchymal tumors, high-grade RCCs with sarcomatoid change may retain PAX8 positivity [13, 14]. PAX2 is a similar protein, but it is more likely positive in malignant mesenchymal tumors including rhabdomyosarcoma and synovial sarcoma, so PAX2 must be cautiously used to differentiate between sarcomas and sarcomatoid RCCs [15]. As it was indicated, a unique feature of the FDCS is the expression of follicular dendritic cell markers like CD21 and CD23 [1–3]. These markers are negative in other malignant mesenchymal tumors. Our FDCS was deeply located in the renal medulla, so another tumor in the differential diagnosis is an invasive urothelial carcinoma, especially a lymphoepithelioma-like variant or sarcomatoid variant [16]. First of all, these UCC variants are unusual in this localization, on the other hand, they retain cytokeratin expression, which is missing from the FDCS of the kidney [16]. Inflammatory cell infiltration is the most characteristic hallmark of the FDCSs. Actually, the lymphocytes are intimately associated with the tumor cells. This feature raises the possibility of an inflammatory myofibroblastic tumor (IMT) that can be developed in the kidney, especially on the ground of renal stones and chronic inflammation [17]. Like FDCS, IMT is built up by spindle-shaped cells with numerous mitotic figures. However, the cytological atypia is moderate in IMT, and the tumor cells are positive with cytokeratin and more importantly with smooth muscle actin. The genetic background of IMT and FDCS is also different, namely IMT harbors *ALK* rearrangements, while FDCS is characterized by mutations or copy number alterations of oncogenes [7, 18]. The former covers *BRAF* V600E mutation which is present in about 20% of the FDCS case, while the latter includes *MDM2* amplification [19], and this genetic alteration leads us to the most important entity in the differential diagnosis, namely the dedifferentiated liposarcoma (DLS). DLS evolves from a well-differentiated liposarcoma, and most commonly, DLS arises in the retroperitoneum [20]. The tumor is composed of spindle-shaped cells with significant pleomorphism [20]. Furthermore, in DLS, a loose and inflammatory background can be observed. Genetically, DLS is characterized by *MDM2* and *CDK4* amplifications that can be studied by immunohistochemistry and FISH [21]. Agaimy and his colleagues found that both MDM2 immunohistochemistry and FISH provided a positive result in FDCS, but the CDK4 was negative in the tumor cells [19]. Additionally, S100 can be occasionally expressed in both DLS and FDCS. Of note, the transition between the well-differentiated and dedifferentiated areas is usually present in surgical resections, but in biopsy samples, the core may contain the dedifferentiated components. Also, somatostatin receptor 2 A (SSTR2A) is a novel diagnostic marker for FDCS, which is extensively expressed by meningioma as well [19]. Regarding our case, the SSTR2A positivity undoubtfully supported the diagnosis of FDCS because meningioma of the retroperitoneum is an anecdotic entity [22]. FDCS is an intermediate-grade tumor [2], with up to 40% experiencing local recurrence and 25% metastatic spread [4]. Surgical removal of the tumor can be curative in early stages [19]. In this case, the patient underwent radical nephrectomy with no adjuvant systemic treatment, and after 91 months of follow-up, she remains alive and tumor-free. Recent findings indicate that a significant portion of FDCS cases express PD-L1 [19], suggesting potential benefit from immunotherapy targeting the PD1/PD-L1 axis.

In summary, we presented a rare case of follicular dendritic cell sarcoma arising from the renal parenchyma, with detailed morphological features and confirmed *MDM2* amplification by FISH. We also discussed the most important entities in the differential diagnosis. After almost eight years of follow-up, the patient remains alive and tumor-free.

### Electronic supplementary material

Below is the link to the electronic supplementary material.

**Supplementary Material 1: Suppl. Figure 1** Additional microscopic feature of the renal follicular dendritic cell sarcoma. **a** The atrophic and fibrotic renal parenchyma covered the tumor. Some sclerotic glomeruli (arrowheads) were present in the fibrotic tissue (magnification factor of 50x). **b** The tumor cells expressed CD23 almost diffusely (magnification factor of 200x). **c** The CD68 decorated the histiocytes; however, it was weakly expressed by some tumor cells, too (magnification factor of 200x). **d** S100 highlighted only scattered dendritic cells, while the neoplastic cells were negative (magnification factor of 200x)

**Supplementary Material 2: Suppl. Table 1** List of the antibodies applied in this study

## Data Availability

All data generated or analyzed during this study are included in this article. Further inquiries can be directed to the corresponding author.

## Abbreviations

DLS: Dedifferentiated liposarcoma
FDCS: Follicular dendritic cell sarcoma
FISH: Fluorescent in situ hybridization
IMT: Inflammatory myofibroblastic tumor
RCC: Renal cell carcinoma
SSTR2A: Somatostatin receptor 2 A
