# Supplementary figures and images for: Primary follicular dendritic cell sarcoma of the kidney – a case report of a rare tumor with emphasis on diagnostic pitfalls
Source: Diagn Pathol. 2024 Jan 31;19:24. doi: 10.1186/s13000-024-01444-x (PMC10829294; doi:10.1186/s13000-024-01444-x)

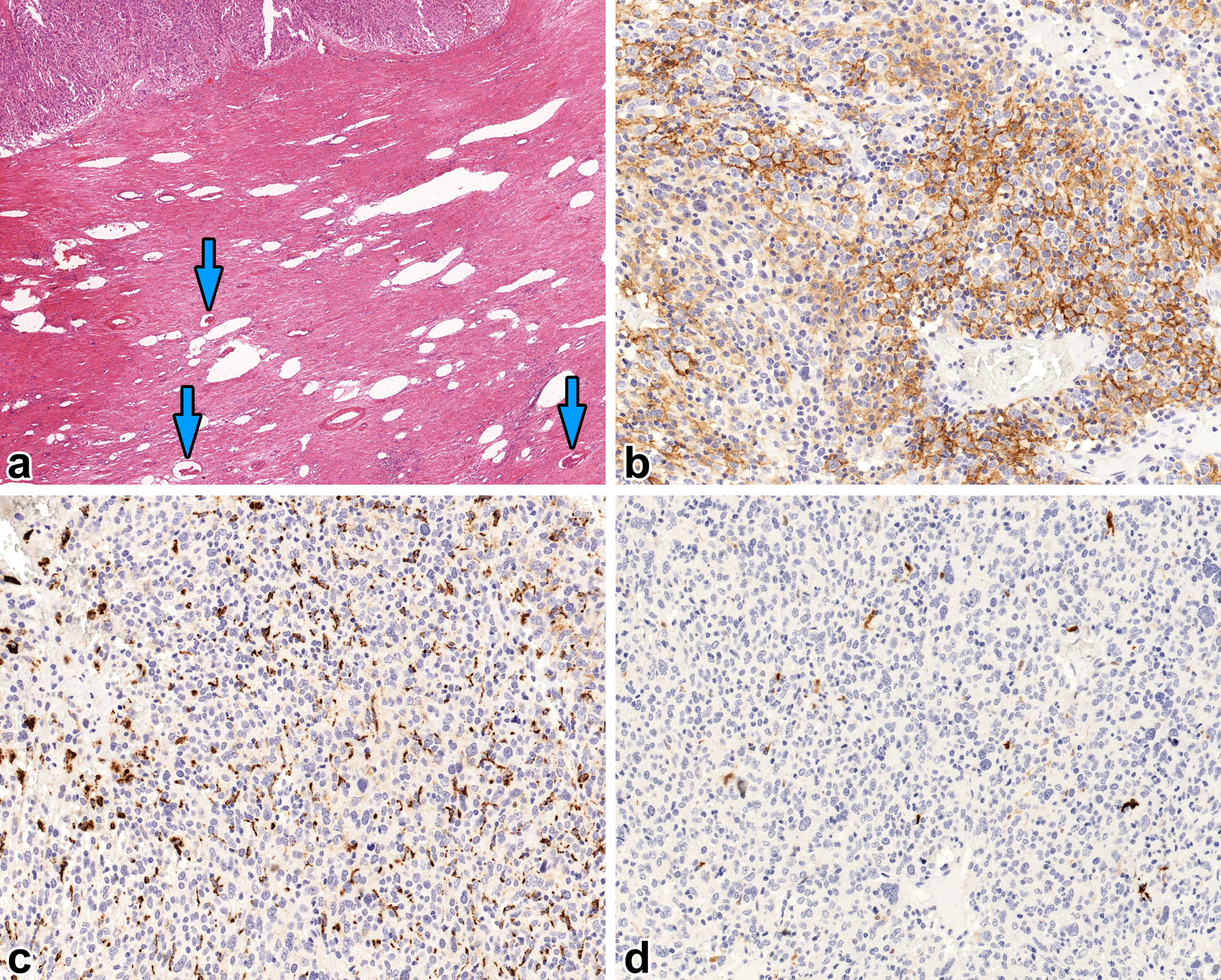

Supplement: Supplementary file 1 — Supplementary Material 1: Suppl. Figure 1 Additional microscopic feature of the renal follicular dendritic cell sarcoma. a The atrophic and fibrotic renal parenchyma covered the tumor. Some sclerotic glomeruli (arrowheads) were present in the fibrotic tissue (magnification factor of 50x). b The tumor cells expressed CD23 almost diffusely (magnification factor of 200x). c The CD68 decorated the histiocytes; however, it was weakly expressed by some tumor cells, too (magnification factor of 200x). d S100 highlighted only scattered dendritic cells, while the neoplastic cells were negative (magnification factor of 200x) [file 13000_2024_1444_MOESM1_ESM.png]
