# Supplementary material for: Primary follicular dendritic cell sarcoma of the kidney – a case report of a rare tumor with emphasis on diagnostic pitfalls
Source: Diagn Pathol. 2024 Jan 31;19:24. doi: 10.1186/s13000-024-01444-x (PMC10829294; doi:10.1186/s13000-024-01444-x)
Supplement: Supplementary file 2 — Supplementary Material 2: Suppl. Table 1 List of the antibodies applied in this study [file 13000_2024_1444_MOESM2_ESM.docx]

| **Antibody** | **Source** | **Clone** | **Dilution** |
| --- | --- | --- | --- |
| CD21 | Cell Marque | EP3093, rabbit monoclonal | 1:100 |
| CD23 | Cell Marque | MRQ-57, mouse monoclonal | 1:500 |
| SSRA2 | BioSB | EP149, rabbit monoclonal | 1:1000 |
| HHV8 | BioSB | 13B10, mouse monoclonal | 1:100 |
| MDM2 | BioSB | BSB-64, mouse monoclonal | 1:50 |
| PAX8 | Cell Marque | MRQ50, mouse monoclonal | 1:400 |
| PAX2 | BioSB | EP235, rabbit monoclonal | 1:100 |
| AE1/AE3 | Cell Marque | AE1/AE3, mouse monoclonal | 1:600 |
| CK7 | Cell Marque | OV-TL 12/30, mouse monoclonal | 1:500 |
| MelanA | Labvision | A103, mouse monoclonal | 1:1500 |
| S100 | Cell Marque | 4C4.9, mouse monoclonal | 1:800 |
| CD68 | Dako | PG-M1, mouse monoclonal | 1:100 |
| SMA | Cell Marque | 1A4, mouse monoclonal | 1:300 |
| TdT | BioSB | EP266, rabbit monoclonal | 1:100 |
| Desmin | BioSB | D33, mouse monoclonal | 1:200 |
| Podoplanin | Cell Marque | D2-40, mouse monoclonal | 1:50 |
